# Physicochemical Properties and Antioxidant Activities of Luteolin-Phospholipid Complex

**DOI:** 10.3390/molecules14093486

**Published:** 2009-09-09

**Authors:** Keyong Xu, Benguo Liu, Yuxiang Ma, Jiquan Du, Guanglei Li, Han Gao, Yuan Zhang, Zhengxiang Ning

**Affiliations:** 1 College of Light Industry and Food Science, South China University of Technology, Guangzhou 510640, China; E-mails: kyxky@163.com (K.X.); fezhning@scut.edu.cn (Z.N.); 2 School of Food Science, Henan Institute of Science and Technology, Xinxiang 453003, China E-mails: jiquan19860303@163.com (J.D.); lgl70@hist.edu.cn (G.L.); gh@hist.edu.cn (H.G.); zy982@163.com (Y.Z.); 3 College of Grain and Food, Henan University of Technology, Zhengzhou 450052, China E-mail: myx366@163.com (Y.M.)

**Keywords:** luteolin, phospholipid, flavonoid, physicochemical property, antioxidant

## Abstract

A luteolin and phospholipid complex was prepared to improve the lipophilic properties of luteolin. The physicochemical properties of the complex were analyzed by ultraviolet-visible spectrometry (UV), infrared spectrometry (IR), X-ray diffractometry (XRD) and differential scanning calorimetry (DSC). The results showed that luteolin and phospholipid in the complex were joined by non-covalent-bonds and did not form a new compound. It was found that the complex was an effective scavenger of DPPH radicals, with an IC_50_ value of 28.33 μg/mL. In the Rancimat antioxidant test using lard oil as substrate, the complex also showed the strong antioxidant activity.

## 1. Introduction

**Figure 1:**
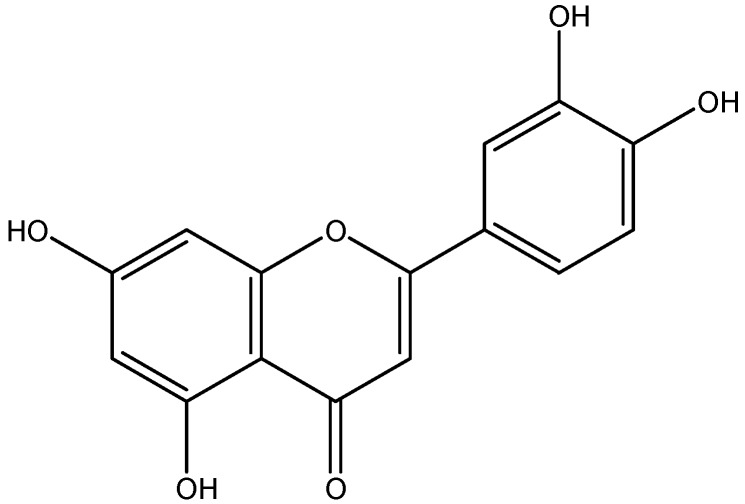
Chemical structure of luteolin.

Luteolin (Figure 1), abundant in celery, green pepper, parsley, perilla leaf and chamomile tea, is one of the most common flavones [1]. It is thought to play an important role in the human body as an antioxidant, a free radical scavenger, an agent in the prevention of inflammation, a promoter of carbohydrate metabolism, and an immune system modulator. These characteristics of luteolin are also believed to play an important part in the prevention of cancer. Multiple research experiments describe luteolin as a biochemical agent that can dramatically reduce inflammation and the symptoms of septic shock [2]. But the low solubility of luteolin in oil results in its poor permeation across the intestinal epithelial cells and the gastrointestinal tract. Phospholipids are an important component of cell membranes, keeping cell membrane fluidity and for treating hepatic disorders. Bombardelli *et al.* have studied the preparation and the biological activity of complexes of several natural principles such as silimarin and aescin with phospholipids [3]. The complexes exhibited phamacological activities significantly greater than those observed for the free constituents. It is expected that luteolin combined with phospholipids might result in an improvement of the lipophilic properties of luteolin. In this study, a complex of luteolin and phospholipid was prepared and the physicochemical properties and antioxidant activities of the complex were investigated.

## 2. Results and Discussion

### 2.1. Luteolin–phospholipid complex

In this study, we tried to prepare the luteolin-phospholipid complex to improve the lipophilic properties of luteolin. We prepared the complex with different ratios of phospholipid and luteolin, such as 0.5, 1, 1.5, 2, 2.5 and 3. The results showed that when the ratio was lower than 2.5, the stability of the complex was worse.Tto get the best complex and use the smallest quantity of phospholipid, we finally prepared a luteolin-phospholipid complex with a 2.5 ratio of ingredients. The obtained complex was used for the subsequent structural analysis and antioxidant assays.

### 2.2. UV and IR analysis

**Figure 2:**
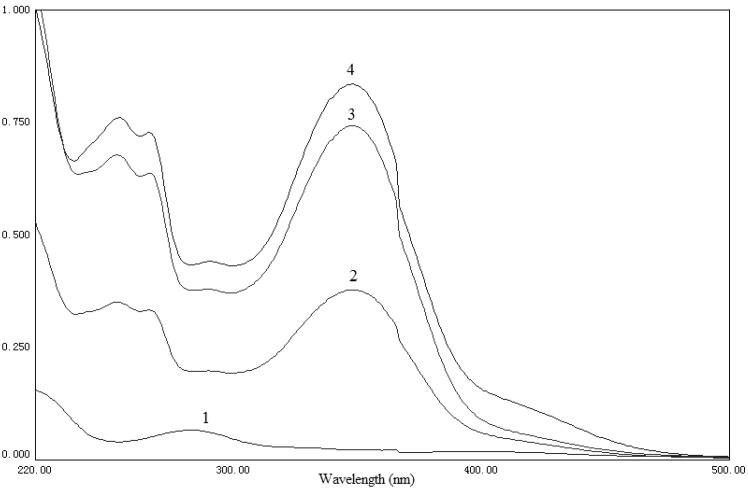
UV spectra of phospholipid (1), physical mixture of luteolin and phospholipid (2), luteolin-phospholipid complex (3) and luteolin (4).

**Figure 3:**
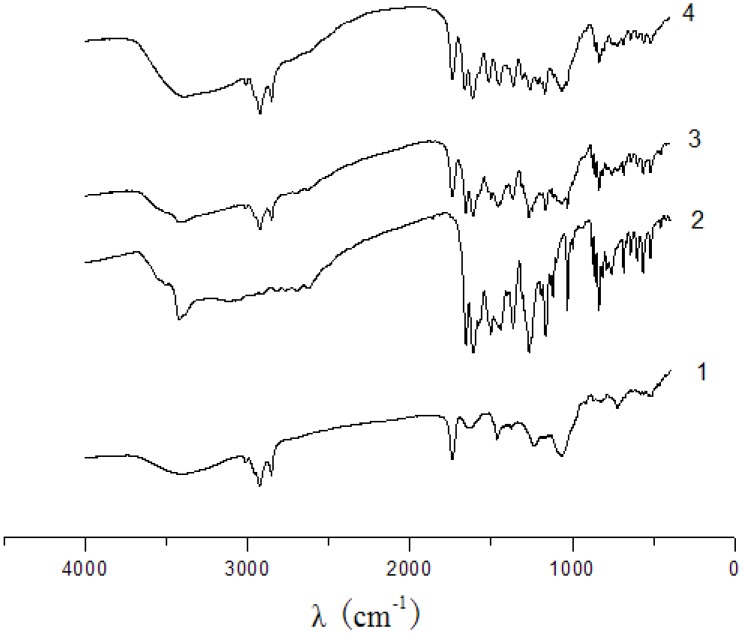
IR spectra of phospholipid (1), luteolin (2), physical mixture of luteolin and phospholipid (3), luteolin-phospholipid complex (4).

The UV spectra of phospholipid, luteolin, their physical mixture and the complex are shown in Figure 2. There was no difference between the physical mixture and the complex. The characteristic absorption peaks of luteolin (254, 266, 348 nm) were still present. The infrared spectra of phospholipid, luteolin, their physical mixture and the complex are shown in Figure 3. There was no significant difference between the physical mixture and the complex and no new significant peaks were observed. The spectra of the physical mixture and the complex showed an additive effect of luteolin and phospholipid, in which the characteristic absorption peaks of phospholipid and luteolin were still present at 1,741 cm^-1^ and 1,657 cm^-1^, respectively. These observations suggest that some weak physical interactions between luteolin and phospholipid took place during the formation of the complex.

### 2.3. XRD analysis

**Figure 4:**
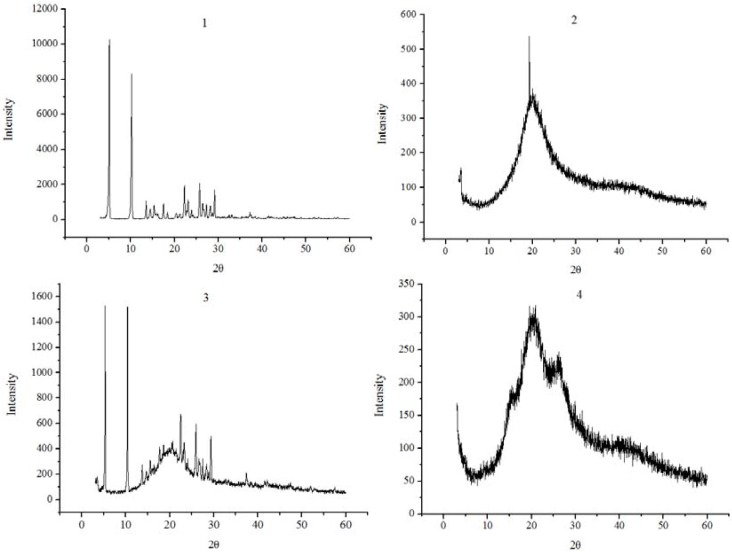
X-ray diffraction patterns of luteolin (1), phospholipid (2), physical mixture of luteolin and phospholipid (3), luteolin-phospholipid complex (4).

The powder X-ray diffraction patterns of luteolin, phospholipid, their physical mixture and the complex are shown in Figure 4. The powder diffraction pattern of luteolin displayed sharp crystalline peaks, which is the characteristic of an organic molecule with crystallinity. In contrast, phospholipid showed an amorphous state lacking crystalline peaks. Compared with that of the physical mixture, the crystalline peaks had disappeared in the complex. This suggested that luteolin in the phospholipid lipid matrix was either molecularly dispersed or in an amorphous form. However, some crystalline drug signal was still detectable in the physical mixtures of phospholipid and luteolin.

### 2.4. DSC analysis

**Figure 5:**
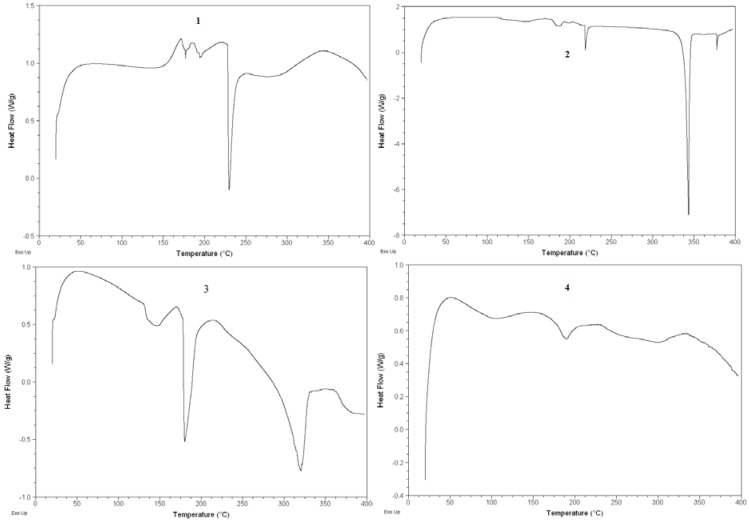
DSC curves of luteolin (1), phospholipid (2), physical mixture of luteolin and phospholipid (3), luteolin-phospholipid complex (4).

Figure 5 shows the DSC curves of phospholipid, luteolin, their physical mixture and the complex. The DSC curve of luteolin showed an endothermal peak with an onset temperature at about 320 °C, which was attributed to the melting of luteolin. The DSC curve of the physical mixture mainly showed the effect of luteolin and phospholipid. But the DSC curve of the complex mainly showed the effect of phospholipid, in which the characteristic endothermal peaks of luteolin disappeared. According to the report of Lasonder and Weringa [4], it was considered that luteolin had been completely dispersed in phospholipid and the components would just have some interaction through hydrogen bonds or van der Waals force.

### 2.5. DPPH radical scavenging activity

**Figure 6:**
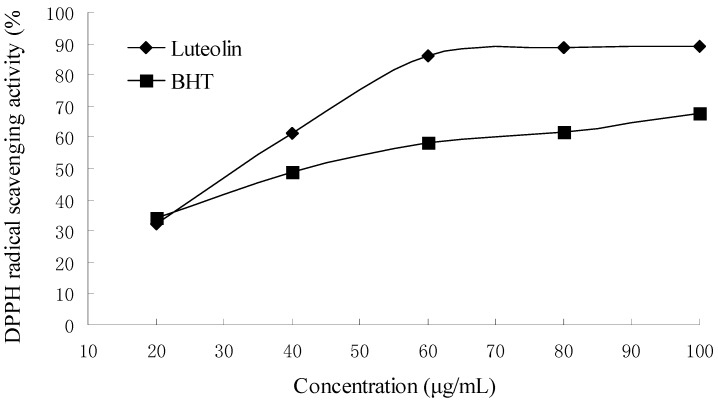
DPPH radical scavenging activity of BHT and luteolin-phospholipid complex.

The DPPH radical is a stable organic free radical with an adsorption band at 517 nm. It loses this adsorption when accepting an electron or a free radical species, which results in a visually noticeable discoloration from purple to yellow. In this study, the high DPPH radical scavenging activity of BHT and the complex was observed in a concentration dependent manner (Figure 6). The complex (IC_50,_ 28.33 μg/mL) was more active than BHT (IC_50_, 42.62 μg/mL). The high DPPH radical scavenging activity of the complex is attributed to the presence of luteolin.

### 2.6. Antioxidant activity in Rancimat test

**Figure 7:**
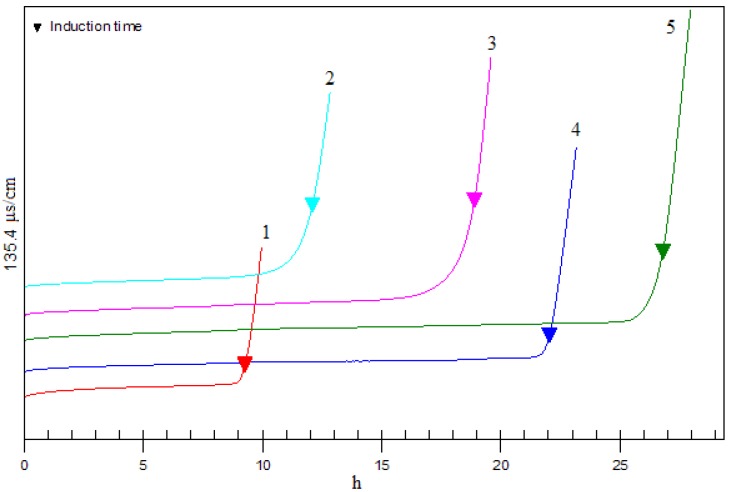
Rancimat antioxidant activity of antioxidant activity of control (1), 0.02% complex (2), 0.04% complex (3), 0.02% BHT (4) and 0.06% complex (5).

In the Rancimat method, the sample is exposed to a stream of air at temperatures from 50-220 °C. The volatile oxidation products (chiefly formic acid) are transferred to the measuring vessel by the air stream and absorbed there in the measuring solution (distilled water). When the conductivity of this measuring solution is recorded continuously, an antioxidation curve is obtained whose point of inflection is known as the induction time, which provides a good characteristic value for the oxidation stability. As shown in Figure 7 the induction times of control, 0.02% complex, 0.04% complex, 0.06% complex and 0.02% BHT were 9.27 h, 12.10 h, 18.9 h, 26.8 h, and 22.8 h, respectively. With the increase of the addition, the performance of the complex could compare with that of BHT. The result showed the possibility that the complex could be used in oils or lipophilic foods.

## 3. Experimental Section

### 3.1. Materials and chemicals

Luteolin (Purity 98%) was purchased from Shaanxi Huike Botanical Development Co., Ltd (Xi’an, Shaanxi, China). 1,1-Diphenyl-2-picrylhydrazyl (DPPH) was purchased from Sigma Chemicals Co. (St. Louis, MO, USA). Phospholipid from soya bean (lecithin) was the product of Sangon Corporation (Shanghai, China). Other chemical were of analytical grade.

### 3.2. Preparation of luteolin-phospholipid complex

Luteolin (100 mg) and phospholipid (250 mg) were dissolved in tetrahydrofuran (100 mL) and stirred for 5 h. After tetrahydrofuran was removed, the residue was collected and ground. The resultant yellow power was collected as luteolin-phospholipid complex.

### 3.3. UV and IR analysis

UV analysis was performed on a TU-1810PC UV-visible spectrophotometer (Purkinje, Beijing, China) and IR analysis was performed on a TENSOR 27 infrared spectrophotometer (Bruker, Karlsruhe, Germany) by the KBr method.

### 3.4. X-ray diffractometry (XRD)

Monochromatic Cu Ka radiation (wavelength = 1.54056 Å) was produced by a D/MAX 2500V/PC X-ray diffractometer (Rigaku Americas Corporation, Tokyo, Japan). The powders of samples were packed tightly in a rectangular aluminum cell. The samples were exposed to the X-ray beam from an X-ray generator running at 36 kV and 20 mA. The scanning regions of the diffraction angle, 2θ, were 5–60°. Duplicate measurements were made at ambient temperature. Radiation was detected with a proportional detector.

### 3.5. Differential scanning calorimetry (DSC)

The samples sealed in the aluminum crimp cell were heated at the speed of 10 °C/min from 0 to 400 °C in the atmosphere of nitrogen (Q200, TA Corporation, New Castle, Delaware, USA). The data were recorded and processed by Universal Analysis 2000 software (TA Corporation, New Castle, Delaware, USA).

### 3.6. DPPH radical scavenging assay

DPPH radical scavenging assay was done according to a published method [5]. Briefly, DPPH solution (2 mL, 0.2 mmol/L in ethanol) was incubated with different concentrations of the samples. The reaction mixture was shaken and incubated in the dark for 30 min, at room temperature. And the absorbance was read at 517 nm against ethanol. Controls containing ethanol instead of the antioxidant solution, and blanks containing ethanol instead of DPPH solution were also made. The inhibition of the DPPH radical by the samples was calculated according to the following formula:

$$\mathrm{DPPH}\;\mathrm{scavenging}\;\mathrm{activity}\;(\%)=\frac{\mathrm{Abs.}\;\mathrm{of}\;\mathrm{control}-(\mathrm{Abs.}\;\mathrm{of}\;\mathrm{simple}-\mathrm{Abs.}\;\mathrm{of}\;\mathrm{blank})}{\mathrm{Abs.}\;\mathrm{of}\;\mathrm{control}}\times \mathrm{100}\%$$

The percentage of DPPH radical scavenging activity was plotted against the sample concentration to obtain the IC_50_, defined as the concentration of sample necessary to cause 50% inhibition.

### 3.7. Rancimat test

The antioxidant activities of BHT and the luteolin-phospholipid complex were measured on a 743 Rancimat analyzer (Metrohm Corporation, Herisau, Switzerland) according to the procedure followed by Proestos *et al*. [6]. Samples of lard oil (3 g) containing 0.02% of BHT and 0.02%, 0.04%, 0.06% of luteolin-phospholipid complex were subjected to oxidation at 110 °C (air flow 20 L/h), respectively. Induction periods, IP (h), were recorded automatically.

## 4. Conclusions

Using tetrahydrofuran as a reaction medium, luteolin and phospholipid were dissolved in the medium, and after the organic solvent was removed, the luteolin–phospholipid complex could be obtained. Using UV, IR, XRD and DSC, it could be concluded that luteolin and phospholipid in the complex were joined by non-covalent-bonds, and did not form a new compound. The obtained complex showed strong antioxidant activity and could be used in oils or lipophilic foods.
